# Prevention and Management of COVID-19 in Patients with Dementia Living at Home: Experience from China

**DOI:** 10.14336/AD.2021.0403

**Published:** 2021-06-01

**Authors:** Renjun Lv, Qingqing Yin

**Affiliations:** Department of Geriatric Neurology, Shandong Provincial Hospital Affiliated to Shandong First Medical University, Jinan, Shandong, 250021, China.

**Dear Editor**

Since the initial reports of Coronavirus Disease 2019 (COVID-19) in December 2019, pneumonia caused by severe acute respiratory syndrome coronavirus 2 (SARS-CoV-2) has spread rapidly worldwide, causing a global pandemic [1]. As of the beginning of February 2021, the cumulative incidence COVID-19 has totaled over 102.1 million reported cases and over 2.2 million deaths globally since the start of the pandemic (/www.67who.int/emergencies/diseases/novel-coronavirus-2019). The elderly are a fragile population to be considered at the onset of any natural disaster and crisis, such as the outbreak of COVID-19. Globally, more than 50 million individuals are diagnosed with dementia, and a new case is diagnosed every three seconds. Dementia has become an epidemic disease in the aging society [2]. The COVID-19 epidemic has had a significant impact on the physical and mental health of the public. Older individuals with cognitive disorders are not only more susceptible to viral infection but are also more prone to psychological stress [3,4]. The double-hit of dementia and epidemic of COVID-19 has attracted great attention for the care of individuals with dementia.

China has the largest elderly population in the world and the COVID-19 outbreak has exerted a huge impact on public health in China. Because dementia patients do not fully comprehend prevention and control measures and rely heavily on nursing care, they are not only a susceptible population but are also more likely to become new spreaders of the disease once infected. In order to ensure more careful management of patients with cognitive impairment in China during the period of epidemic prevention and control, the Ministry of Civil Affairs formulated a series of “Expert Recommendations on Mental Health and Psychosocial Support for Persons with Cognitive Disorders and their Caregivers during the COVID-19 Outbreak” (http://rs.yiigle.com/CN113661202002/1186144.htm). Currently, China has contained the epidemic and residential and business activities have begun to return to normal. Based on the guidelines issued by relevant state departments, this article summarizes the main experience from China in the prevention and management of COVID-19 among elderly patients with dementia living in a home-based setting. We believe that our learning experience in China will ease tensions worldwide in the face of the COVID-19 pandemic and reduce the risk of compromising the quality of life of dementia patients and their caregivers.

Following the COVID-19 outbreak and based on the characteristics of elderly individuals living at home, the government, through the media, networking, and door-to-door canvassing, intensified the diffusion of information regarding epidemic prevention and disease control in order to guide elderly individuals and their families to scientifically understand disease transmission, enhance awareness of prevention and control, and provide guidelines to improve protection from infection. Medical and health institutions of all levels and types should fully grasp the scope of health information needed for this diverse population of elderly individuals living alone, empty nesters, elderly people in rural China whose children have left to work in other areas (known as the ‘left behind’ elderly), and patients with disabilities or experiencing various chronic diseases. Such institutions should also pay specific attention to their health status in order to ensure successful health management. Furthermore, it is necessary to recognize the importance of the role of village doctors in achieving epidemic prevention and control of the left-behind elderly (www.gov.cn/xinwen/2020-01/28/content5472793.htm). Based on the guidelines for the prevention and control of the elderly at home, family members are required to assist people with dementia and should assume the following responsibilities: storing of epidemic prevention supplies such as thermometers, masks, and disinfectants; opening windows to increase indoor ventilation to 2-3 times a day, for 20-30 minutes each time; and maintaining normal daily life habits, such as ensuring adequate sleep, a light diet, and balanced nutrition. Dementia patients with chronic lung disease and cardiovascular diseases should wear masks under the professional guidance of doctors. For patients with severe dementia who are dependent on home care, caregivers should pay attention to their own health. Caregivers should pay attention to limiting outings, and if they must go out to ensure adequate self-protection.

The clinical staff of medical institutions at all levels, old-age services, pension institutions, and integrated medical and nursing institutions should provide prevention and education materials based on the specific characteristics of attending patients and their high-risk groups. The public should be given relevant scientific knowledge and resources free of charge in order to enhance public awareness of mental health, the residents' awareness of the prevention and treatment of Alzheimer's disease, and to reduce prejudice and discrimination (www.gov.cn/zhengce/zhengceku/2020-09/11/content_5542555.htm). Psychiatric, Neurology, and Geriatrics departments of psychiatric hospitals or general hospitals, should rely on clinical consultation and cooperation and send experts from the national health care system for assistance, training, and guidance in formulating structured protocols for management and treatment of community- or village-based patients with dementia. The national grassroots health system carries out prevention and intervention services for patients with senile dementia with the help of medical and other service models. For elderly individuals diagnosed with mild cognitive impairment, community (village) general practitioners should organize regular cognitive training sessions aimed at preventing and reducing the incidence of Alzheimer's disease. For patients diagnosed with senile dementia, community doctors should carry out training for the patient’s families and caregivers to improve the intervention rate and improve their quality of life.

Encouraging the grassroots health system to purchase drugs for the treatment of Alzheimer's disease will increase the accessibility of grassroots drugs. When elderly individuals with underlying diseases require prolonged chronic treatment, they should not stop taking medicine without any medical authorization. Patients should attend local community health services to seek medical treatment and for prescription of medicines or obtain comprehensive prescriptions only after medical evaluation. Further, the number of prescription medications should be reduced, and sufficient standards of self-protection should be maintained when seeking medical treatment. Family doctors can also obtain medicines on behalf of patient’s families and should take full advantage of the services provided by the grassroots health system to strengthen the daily management of patients with chronic diseases (www.gov.cn/zhengce/zhengceku/2020-09/11/content_5542555.htm). During the COVID-19 outbreak, restricting outings and gatherings, requiring wearing of masks and frequent washing of hands are still important measures for epidemic prevention and control. Unfortunately, these public health interventions have had a tremendous impact on the well-being of older individuals with dementia and on their caregivers and may ultimately worsen the behavioral and psychological symptoms of dementia [5]. Thus, the epidemic prevention headquarters of each district and county should set up a local township or neighborhood-based comprehensive mental health management team (Integrated management team). The duties of the Integrated management team are described below.

The integrated management team should arrange personnel to intensify the frequency of regular visits to patients with severe mental disorders at home. For patients with suspected symptoms of fever or pneumonia, the comprehensive management team should promptly send them to the nearest fever clinic for treatment and assessment. For a COVID-19 diagnosis or other suspected infection, patients should be admitted to hospital for treatment. The receiving medical institutions should be informed of the patient's past history of mental illness and current treatment. In addition, the integrated management team should pay close attention to the medication compliance of patients with severe mental disorders at home. Contact between patients and their families can be via web-based video and telephone communication, and initiatives to maintain high quality community care and services should be designed and implemented.

For patients living in closed management areas, a local management team should take initiative of understanding their patients’ medication needs, assist in mailing drugs to mental health institutions, or delivering drugs to the grassroots health system. Further, the comprehensive management team should collect drugs from dispensaries and provide door-to-door delivery to help patients maintain continuous drug treatment (www.gov.cn/xinwen/2020-02/19/content_5480748.htm). It is necessary to implement strict and precise protocols with technical guidance from psychiatrists and community prevention personnel. For patients experiencing obvious mental symptoms, irritability, or uncontrolled behavior, the comprehensive management team can contact mental health institutions directly by means of telephone and network consultations, and psychiatrists should provide telemedicine services to home-based patients. For patients in need of emergency care, the integrated management team shall assist in transferring them to mental health institutions for medical treatment.

During the COVID-19 pandemic, medical and health institutions at all levels announced the continuation of medical services in a timely manner and aimed to reduce the waiting time required for patients' families to receive consultations regarding patients using the Internet and/or telephone appointments to ensure more suitable and convenient medical treatment for the elderly in need. Active participation in an "Internet+Health for the Elderly" service (www.retow.com/product/hisDetail/), allows online counseling and other approaches to provide health guidance and services for patients. Strengthening contract services for family doctors and establishing interactive communication channels between family doctors and the elderly and their caregivers by using information means such as Wechat and mobile phone applications has also shown to be a successful approach.

It is government responsibility to ensure the quality of life of dementia patients isolated at home during the COVID-19 pandemic, by strengthening the integration of different policies and practices to effectively guarantee continued essential and medical care. Timely temporary assistance should be given to dementia patients with special difficulties who meet the requirements. Timely and full payment of nursing subsidies, pension service subsidies, and allowances should be provided for the elderly who meet the requirements (www.cncaprc.gov.cn/llxw/191262.jhtml). Improved community-based management measures, with the establishment of centralized sale and distribution of materials for community living, coordination of qualified suppliers, distribution and services of large supermarkets or community-based markets should be guaranteed, and the sale of essential basic foods such as rice grains and oils, boiled eggs, milk and fruits and vegetables should be centralized, to ensure adequate supply of materials for the daily living of community-dwelling individuals (www.gov.cn/zhengce/zhengceku/2020-04/16/content_5503261.htm). A centralized distribution area of express delivery (takeout) should be set up in the community and organized according to the residential address to avoid small-scale gathering of community residents: Pick-up or collection services can be provided in places where conditions permit, and special personnel can be organized to distribute supplies in batches.

Altogether, the epidemic situation in China has been effectively controlled, and the measures taken for home dementia patients have achieved undeniable results. However, there have also been some difficulties and challenges. For example, a problem with the insufficient supply of epidemic prevention materials has emerged. Further, the development of home care services for the elderly in the community has been currently interrupted. Community-based management teams, community workers, caregivers, nannies, and other personnel are lacking, and some community home care service facilities are closed, and thus community home care services for the elderly cannot operate normally.

The sudden outbreak of the COVID-19 pandemic has exposed shortcomings in the development of health services for the elderly in our country. In the future, when establishing a health service system for patients with senile dementia, attention should be paid to socialized cooperation to ensure a management model allowing coordination and communication among different government departments, the efficient operation of grassroots communities, and active participation in local market and social organizations, so as to achieve more comprehensive health services for dementia patients.
